# Books and Authors

**Published:** 1908-01

**Authors:** 


### BOOKS AND AUTHORS.

---

**The Practical Medicine Series of Year Books.** Ten volumes. Issued under the general editorial charge of **Gustavus P. Head**, M.D., Professor of Laryngology and Rhinology in the Chicago Post-Graduate Medical School. Vols. 1, 2 and 3, General Medicine, Surgery, Eye, Ear, Nose and Throat. Series 1907. Chicago: The Year Book Publishers. (Price, $1.25, $1.50, $2.00; entire series, $10.00.)

Volume I. *General Medicine.*—Something over one hundred pages in this number are devoted to the consideration of pulmonary tuberculosis, in the course of which valuable hints are given with reference to all clinical phases of the disease,—diaguosis, the employment of tuberculin, the opsonic index, and the serums being especially dealt with. The other diseases of the lungs and affections of the pleura next follow, all being liberally considered. Diseases of the heart and bloodvessels, too, are given ample treatment; then diseases of the blood-making organs receive due attention. Rheumatism, also, has an excellent handling and, finally, diseases of the kidney are set forth. These are the salient features of the book, though there are several other topics dealt with.

Volume II. *General Surgery.*—This entire volume is prepared by John B. Murphy, and he has filled it with good things. An article by J. L. Thomas commenting on modern fashions in surgery is liberally abstracted; considerable attention is given to operative technic; anesthesia receives much attention, and the whole subject of mechanical therapeutics is gone over. The work of the Mayo Brothers in stomach surgery and Murphy's own work in intestinal surgery furnish interesting and instructive reading. The abstracting of the whole volume has been made with discrimination, and affords valuable information in a nutshell.

Volume III. *The Eye, the Ear, the Nose and Throat.*—This is an exceedingly instructive volume and, like the other number just noticed, has been judiciously abstracted. It is a book that specialists may examine with profit, while to the general practitioner who wishes to keep pace with the progress of medicine it is indispensable.

---

**Human Anatomy,** including Structure and Development and Practical Considerations. Edited by **George A. Piersol,** M.D., Professor of Anatomy in the University of Pennsylvania. Royal octavo, pp. 2108. With 1734 illustrations, of which 1522 are original and largely from dissections by John C. Heisler, M.D., Professor of Anatomy in the Medico-Chirurgical College. Philadelphia and London: J. B. Lippincott Company. (Price, $7.50.)

At first thought one is liable to ask if there is need of or room for another anatomy; and the answer if given before an examination is made of this work is liable to be in the negative. But once to examine is to be convinced. Whether this work is judged by the number and quality of its illustrations; or the excellence of its descriptive text; or, again, by the voluminosity of its pages, these one or all serve to give the book an individuality that makes it a superb exposition of the science of anatomy as at present understood. Moreover, the editor himself, Piersol, is recognised as one of the foremost anatomists that America has produced; he teaches anatomy in one of the first American medical schools, and he has associated with him in the preparation of this work a group of experts that cannot be excelled. These are conditions that conspire to the successful making of a splendid book like this, and the result is all that can be desired.

Here, then, is a treatise that comprises over two thousand pages, containing seventeen hundred and thirty-four illustrations of which over fifteen hundred are original, the latter being largely from dissections made by John C. Heisler, one of the staff of contributors. Add to this the superb workmanship of the publishers, and it would be difficult to conceive a more perfect combination of skill and science, from which to evolve such a book.

It may be remarked, further, as a matter of national pride that this is the first entirely American anatomy in text, illustrations, and printing, and as such it may well appeal to Americans as a distinct novelty even in American medicine. Finally, it is doubtful if more labor or money has been expended heretofore, on any single volume relating to the literature of medicine.

It is not necessary to accord such a book a detailed review; it would be quite impossible within reasonable magazine limits; besides it is doubtful if the reader would be patient under such an affliction. It is a work that must be seen to be appreciated; its beautiful engravings, many in color, are quite beyond adequate pen descriptions, while the whole scheme of the book is stamped with artistic and scientific beauty and value.

---

**Diseases of Infancy and Childhood.** Their Dietetic, Hygenic, and Medical Treatment. A textbook designed for practitioners and students in medicine. By **Louis Fischer,** M.D., Visiting Physician to The Willard Parker and Riverside Hospitals, New York. With 303 text illustrations, several in colors, and twenty-seven full-page half-tone and color plates. 979 royal octavo pages. Philadelphia: F. A. Davis Company. (Cloth $6.50; half morocco $8.00, net prices.)

This book is divided into twelve parts: I. The newborn infant. II. Abnormalities and diseases of the newly-born. III. Feeding in health and disease. IX. Disorders associated with improper nutrition and diseases of the alimentary tract. V. Diseases of the heart, spleen, liver, pancreas, peritoneum and genitourinary tract. VI. Diseases of the respiratory system. VII. The infectious diseases. VIII. Diseases of the blood, lymph nodes, and ductless glands. IX. Diseases of the nervous system. X. Disease of the eye, ear, skin and abnormal growths. XI. Diseases of the spine and joints. XII. Miscellaneous.

This is an excellent classification and indicates a familiarity on the part of the author with the clinical pictures of disease.

Knowledge of a subject by an author is indicated by the orderly manner in which he deals with it. This author lays great stress on diagnosis which is another good indication of his ability. His experience as a teacher of the diseases of children at the New York Post-graduate Medical School and Hospital has stood him in good stead as an author.

Not by any means the least important part of this book is the illustrations. Most of the articles are illustrated clinically or pictorially, one or both, and the pictures are for the most part from original photographs or drawings. The value of good illuminations of the text in a work on diseases children is to be specially emphasised here, because they aid more than in any other treatise, unless it be one on surgery.

As a last word we speak of the importance the author gives to pathology. He has given bacteriology, too, its proper consideration; indeed, laboratory aids to diagnosis have been invoked wherever they could be made serviceable. It is one of the most useful books of the year and will take a high place in the literature of pediatrics.

---

**Gynecology and Abdominal Surgery.** Edited by **Howard A. Kelly,** M.D., Professor of Gynecologic Surgery at the Johns Hopkins University, and Charles P. Noble, M.D., Clinical Professor of Gynecology at the Woman's Medical College, Philadelphia. Imperial octavo, vol. 1, pp. 851. Illustrated by Herman Becker and others.

This remarkable work, appearing now in its first volume, promises to supplement the literature of gynecology, though already somewhat over-crowded, in a useful way. The editors are experienced men and so are the other contributors, hence the material as might be anticipated, is of the better sort. We are pleased to observe the coupling of gynecology and abdominal surgery in the title of the book, as well as to read in the opening paragraph of the preface a manly defense of gynecology against the aspersions of some so-called general surgeons. The fact is that gynecology set the pace in all surgery of the lower cavities of the body, and established a technic so simple and perfect for surgery of the pelvis and abdomen that comparatively few general surgeons have approached it. Hence there is little wonder that the mortality rates of the gynecological surgeon are so much lower in this class of surgery.

The attention is arrested at once upon opening this book by the fact that the classification is unusual, it being adapted to the particular needs of the general practitioner, and to aid the general surgeon, who, as a matter of course, is not so familiar with gynecologic methods. Another striking feature of this work is a section devoted to medical gynecology, which some have supposed to be a "lost art." Again, it is a notable point that certain obstetrico-gynecologic topics are introduced, which include such obstetric diseases as require operative treatment.

Finally, and one is almost tempted to say most importantly, is the question of the illustrations. Like all other medical works, Kelly has written or taken part in, this one is superbly illustrated. The drawings of operative procedures are true to nature and depict the work being done with almost as much benefit as if it were performing in the clinic.

---

**Diagnostics of Diseases of Children.** By **LeGrand Kerr,** M.D., Professor of Diseases of Children at the Brooklyn Postgraduate Medical School. Octavo of 542 pages, illustrated. Philadelphia and London: W. B. Saunders Company, 1907. (Cloth, $5.00 net; half morocco, $6.50 net.)

The literature of diagnostics is being enriched with great rapidity at the present time. Pretentious works on medical and surgical diagnosis referring especially to adults have issued recently; or, to be more specific, several such have appeared during the year just ended. But it has remained for Kerr to become the author of the most important book devoted exclusively to the diagnosis of diseases of children that has fallen under our eye.

It is one thing, however, to discourse of the diagnosis of disease in the grown person, but quite another to interpret the signs of ill-health in the infant or young child. It is as much like translating the æneid of Virgil on the one hand, and attempting to decipher the hieroglyphics of a Luxor column on the other. Mr. Welland Strong, in the "trip to Chinatown" is made to say, in effect, that a horse doctor has the advantage over physicians in general, because his patients can't talk back at him. However, that may be regarding "horse doctors," most of us consider it a great disadvantage when our patients "can't talk back at us," as in the case of infants or very young children.

One of the strong points in this book is the author's methods of interpreting the symptoms presented by these little patients. He has invoked the aid of photography to good purpose in depicting the meaning of these symptoms, and his whole treatise teems with clinical information of value to student and practitioner, senior or junior.

---

**A Textbook of Pathology.** By **Francis Delafield,** M.D., LL.D., and **T. Mitchell Prudden,** M.D., LL.D Eighth Edition. Octavo, 1,075 pages, illustrated by thirteen full-page plates and by 650 line and half-tone cuts. William Wood & Co., New York. Muslin, $5.50; leather, $6.50, net prices.)

This work needs no introduction to the medical profession of America, for it has passed already to honors which belong only to a veteran; indeed, it may justly be termed a classic. The book-shelves that fail to contain "Delafield and Prudden" in one or all of its editions, are poorly equipped for pathological study or reference.

As might be anticipated, several improvements have been made in this edition, though the general aim and purpose of the work have been retained. The section relating to general pathology has been amplified and the various phases of pathologic physiology given more attention than heretofore. Greater stress, too, has been laid upon the relationships of pathology to the allied phases of biology. Many other changes making for improvement have been incorporated, such as increasing the foot-note references, rearranging the contents, revising the sections on the blood and nervous system, the addition of over one hundred and fifty new illutrations, all incident to the rapid accumulation of data in the ever increasing field of pathology.

It will be observed from all these items, as well as many others which we have not found it essential to mention, that it is the purpose of the authors and publishers to keep the work constantly abreast of the period, through the laborious work of frequent revision, which each time embrace both eliminations and additions. The excellence of the illustrations is a source of pride to every one who appreciates artistic and useful engravings as a method of impressing salient features of textbooks, due in this instance largely to the skilful labors of Dr. Edward Leaming.

As a final thought we may remark that it is doubtful if anything that scientific labor or a liberal outlay of money can produce or obtain, has been omitted in the endeavor to make this one of the most perfect textbooks on pathology that has been issued up to the present moment.

---

**A Textbook of the Practice of Medicine.** By **James Magoffin French,** A.M., M.D. Third edition. Octavo, 1267 pages, illustrated by engravings in the text and by twenty-five full-page plates. New York: William Wood & Co. (Muslin, $5.50; leather, $6.50 net prices.)

The first edition of French's practice attracted so much attention that it was exhausted sooner than could have been predicted by the most sanguine expectations of author or publishers. French was then comparatively unknown except in his own region, but his book lifted him at once into a national fame which soon became international in character. In its notice of the first edition the JOURNAL pointed out among others the following distinguishing features of the work—namely, it is compact, being of less dimensions than most textbooks issued recently,—an important factor for students of medicine; its make-up and general form are excellent; it is terse even to conciseness without being elementary; it deals with only the practical, leaving the theoretical behind; it contains the most modern thought and records the most recent discoveries; it omits case reports and instead deals with more useful material, thus economising space and preserving the patience of the reader; finally, it is illustrated with a few well selected and excellently executed plates and engravings that give force and impact to the text. We also ventured to predict that the treatise would assume an important even permanent place in the literacure of medicine, which it already has done.

In its present edition the book has been rewritten completely and its scheme much expanded, with the result that it is placed among the larger and more important textbooks on medicine. Many new illustrations have been added, some of which are full-page plates in colors, and the work is brought forward to the immediate present, in all its sections. It easily takes its place among the better as well as more popular works on the practice of medicine.

---

**Kirkes' Handbook of Physiology.** Sixth American Edition. Revised by **Charles Wilson Greene,** A.M., Ph.D., Professor of Physiology and Pharmacology in the University of Missouri. Octavo, pp. 723. Illustrated. New York: William Wood & Co. (Cloth, $3.00.)

Beginning as an elementary work this standard handbook has gradually broadened its scope through frequent successive editions until now it takes rank as one of the better works on physiology extant; indeed, it is to be found on the college lists as an almost universally adopted textbook. In the present revision many changes are noted, obsolete material having been excluded and newer text has been substituted.

Several of the chapters have been entirely rewritten, especially those on the blood, circulation, respiration, and the nervous system. Every chapter has been reorganised, and sections entirely reconstructed. The material has been chosen with a view to presenting a student's handbook. The facts of recent research have been incorporated, and the newer explanations of physiological processes have been utilised wherever possible. It has not been the aim of either the reviser or of the publishers to present an encyclopedia of physiology or a textbook for the investigator, but rather to perfect the volume as a medical student's manual which shall keep pace with the present rapid advances of medical education.

It is a pleasure to welcome this old friend in a new and advanced form. The publishers in coöperation with the editor have omitted nothing because of expense, to make this as completely useful a book as possible for both practitioner and student.

---

**A Textbook of Practical Therapeutics.** By **Hobart Amory Hare,** M.D., B.Sc., Professor of Therapeutics and Materia Medica in the Jefferson Medical College of Philadelphia. Twelfth edition, revised to accord with the eighth decennial revision of the U. S. Pharmacopeia. Octavo of 939 pages, with 114 engravings and four colored plates. Philadelphia and New York: 1907. Lea Brothers & Co. (Cloth, $4.00; leather, $5.00; half morocco, $5.50 net prices.)

We have commented heretofore on this work as edition after edition has issued from the press. In this twelfth edition, which has been thoroughly—not perfunctorily—revised, more attention has been paid to materia medica than in the previous issues; one-half of the book is devoted to remedial agents, including drugs non-medicinal agents, and foods, the other half being set apart for the consideration of diseases, with precise directions as to their treatment. Both parts are alphabetically arranged and adequately cross-referenced, thus making it easy to search for information desired. Newer drugs are considered, treating syphilis by the best methods of hypodermic medication is set forth, the value of citric acid for preventing thrombosis in typhoid fever is dealt with, so, too, is the danger of toxemia after the use of chloroform and ether,—in short, nothing seems to have been overlooked that will prove of benefit either to student or practitioner of medicine. In short, again, it comes pretty near to being the ideal textbook on practical therapeutics.

---

**A Textbook of Physiology.** By **Isaac Ott,** A.M., M.D., Professor of Physiology in the Medico-Chirurgical College of Philadelphia. Second Edition. Ilustrated with 393 half-tones, many in colors. Royal octavo. 815 pages. F. A. Davis Company, Philadelphia. Cloth, $3.50.)

The renaissance of physiology is taking place if one may judge by the several works that are appearing on the subject, new and old, almost simultaneously from competent authors and responsible publishers. That it is one of the fundamentals of medicine no one can deny; that it should be mastered as far as possible during the first half of the collegiate course is equally certain. The number of authors in this field indicates an increasing interest in this line of investigation. Time was when Dalton in this country held complete sway; now, no single author covers the territory. Several excellent teachers are competing for recognition, among whom Ott may be given place in the front rank.

The second edition has been increased in size by nearly two hundred and fifty pages, which indicates the rapid development of the science of physiology. The book has not only been enlarged but its entire subject-matter has been carefully overhauled, so to speak, making it an exponent of the latest research. It is the largest single volume textbook on physiology offered to American students by an American author, to whom we commend it as among the better works of its class. It will be appreciated, too, by practitioners as a reference volume.

---

**The Treatment of Fractures.** By **Charles L. Scudder,** M.D., Surgeon to the Massachusetts General Hospital. Sixth edition. Octavo 635 pages, with 854 original illustrations. Philadelphia and London: W. B. Saunders Company, 1907. (Polished buckram, $5.50; half morocco, $7.00 net prices.)

Six editions in seven years tells with considerable force of the estimate placed by the medical profession upon this treatise. Since Hamilton's famous work nothing has been more useful than the book we are attempting to deal with, the distinguishing characteristics of which are its clinical features. Its practical value, therefore, is very great, especially as a guide for the junior practitioner. It tells him how to diagnosticate a particular fracture and then how to treat it, illustrating in most instances the precise form of dressing to employ, always keeping in view simplicity and the greater efficiency of the appliance.

The propriety of operating on certain fractures is detailed, some of which are described; obstetrical fractures of the skull in the newborn are dealt with; many fractures of the bones of the face are described; unreduced dislocations of certain joints, pathological fractures, and old fractures are set forth, and the treatment of ununited fractures is discussed.

The illustrations are not only numerous but of excellent quality and especially are the *x*-ray pictures of unusual clearness as well as of diagnostic value. The price of the book is low when the number and quality of the engravings are taken into consideration.

---

**Modern Medicine.** Its Theory and Practice. In original contributions by American and Foreign authors. Edited by **William Osler,** M.D., Regius Professor of Medicine in Oxford University, England. Assisted by **Thomas McCrea,** M.D., Associate Professor of Medicine and Clinical Therapeutics in Johns Hopkins University, Baltimore. In seven octavo volumes of about 1,000 pages each; illustrated. Volume II. Philadelphia and New York: Lea Brothers & Co. 1907. (Cloth, $6.00; leather, $7.00; half morocco, $7.50, net prices.)

This volume, the second in this great series, deals with the infections, to which also the third volume will be devoted. A resume of the contents of this book may not be out of place. Beginning with an introduction to the study of the infections by Hektoen, then McCrea considers typhoid, typhus, and relapsing fever; Councilman, smallpox and chickenpox; Dock, vaccination; McCollom, scarlet fever and diphtheria; Ruhräh, measles, rubella, the fourth disease, erythema infectiosum, whooping cough and mumps; Lord, influenza; Coleman, dengue; Koplik, meningitis; Anders, erysipelas; Musser and Norris, pneumonia; Pearce, toxemia, septicemia and pyemia; Poynton, rheumatism; Dunbar, cholera; Carroll, yellow fever; Calvert, plague, and Shiga, bacillary dysentery.

It will be seen by this array of eminent authors who deal with a group of important subjects that this volume keeps abreast of the first one in the excellent pace it set. When all the volumes, seven in number, shall have been issued, the entire field of medicine will be covered by the most advanced thought, thus lifting the science up to the highest plain of twentieth century knowledge, and bringing it within the grasp of every physician who would wish to obtain the best literature pertaining to the practice of his art.

**A Manual of Clinical Diagnosis by Microscopical and Chemical Methods.** For Students, Hospital Physicians and Practitioners. By **Charles E. Simon,** M.D., Professor of Clinical Pathology in the Baltimore Medical College. Sixth edition, revised. Octavo, 682 pages, with 177 engravings and 24 colored plates. Lea Brothers & Co., Philadelphia and New York, 1907. (Cloth, $4.00 net.)

This remarkable book has appeared in six successive editions within ten years,—a fact that speaks for its value and for the splendid work of the author. Furthermore, it speaks for the growing interest of the medical profession in methods of exact diagnosis; and for an appreciation of all information, as well as of every author who may aid in arriving at the desired result. Simon has demonstrated his ability as a teacher and his capacity for scientific work. His book began as an extremely modest volume, but it has steadily increased in size, until it has become one of the largest and best of its class.

New material has been introduced and everything not practical has been eliminated until the presnt edition has become as near perfect as it is possible to make a book dealing with an ever progressive subject. The new chapter on the opsonins will attract attention, and the one on the blood will be appreciated for the additions made to it since the last previous edition. Two appendixes (we use the word advisedly) have been included among the improvements of the sixth edition,—one relating to the preparation of culture media and the other representing an outline course in clinical laboratory methods. Illustrations have been used whenever they could be made to accentuate the text, many of which, we may be pardoned for saying, are from the facile brush of Mrs. Simon, the accomplished wife of the author.

---

**Symptomatology and Diagnosis of Disorders of Respiration and Circulation.** By **Prof. Edmund von Neusser,** M. D., Professor of the Second Medical Clinic, Vienna. Authorised English Translation by **Andrew MacFarlane,** M.D., Professor of Medical Jurisprudence and Physical Diagnosis, Albany Medical College. Part I. Dyspnea and Cyanosis. 12mo, pp. 203. New York: E. B. Treat & Co. (Cloth, $1.50.)

The two topics dealt with in this monograph are so closely allied as to be included very properly in the same brochure. They are symptoms of respiratory or cardiac disturbances, organic or neurotic; they indicate real lesions or functional faults, and it is important to determine which, else the treatment may be wrong. This book will aid in the solution of the problem. It consists of a series of lectures by a clinician of much experience, with a vast amount of material at command. Every conceivable form of dyspnea and cyanosis is set forth in detail and with precision. It is a book that will prove useful in the hands of every practitioner called upon to treat diseases of the respiratory tracts, upper or lower, or cardiac maladies, functional or organic. The translator has done an admirable piece of work by converting the text into beautiful English. We could wish the diphthong had been eliminated from the word dyspnea. If hyperemia is spelt without the diphthong why should not dyspnea also be so spelled?

---

**A Textbook of Physiology for Students and Practitioners.** By **William H. Howell,** Ph.D., M.D., Professor of Physiology in the Johns Hopkins University, Baltimore. Octavo, pp. 939. Illustrated. Second edition. Philadelphia and London: W. B. Saunders Company. ($4.00 net.)

Something more than two years ago we were pleased to notice in these columns the first edition of this splendid textbook on physiology. It was reprinted from the first plates three times, making practically four editions in two years. Now, in this second edition, a thorough revision has been made, bringing the subject up to the immediate present, while leaving the scope and size of the work as before.

Howell is an experienced teacher and has kept in mind the needs of students in attendance upon courses of experimental pathology and surgery, as well as those upon clinical medicine. The author has displayed good judgment in what he has included as well as excluded from his treatise,—a fact of no mean import in these days of voluminous medical literature. It is easy to conceive that this textbook is destined to a popularity not exceeded by any other work on physiology, and its adoption as such by the colleges in such large numbers, is an index of the value placed upon it by teachers, as well as students.

---

**The Internal Secretions and the Principles of Medicine.** By **Charles E. de M. Sajous,** M.D., Fellow of the College of Physicians of Philadelphia. Volume II. Octavo, pp. 1099. With 25 illustrations. Philadelphia: F. A. Davis Company. 1907. (Cloth, $6.00.)

In this volume the author continues the publication of his researches into the role played by certain secretions in promoting physiological processes; also in studying the part played by leukocytes in arresting tissue loss, or, as Sajous would have us believe, in building tissue. The entire work is full of ingenious theory as well as much practical thought. It is a work based on arduous laboratory investigation and appeals to the student of biology.

Sajous contends for the principle that immunising medication is the foundation of rational therapeutics, applicable alike to the more virulent and to the more benign diseases. The author's studies relating to the functions of the leukocytes are interesting. If they convert food products, disease germs and broken down cells into tissue cells as claimed, then their part in arresting disease is an important one. There is little doubt that the body is provided with its own defences against disease; the important question to solve is the precise manner in which it is done. The work of Sajous in this direction as set forth in this book becomes of vast importance in the treatment of disease.

---

**Blood Stains.** Their Detection and the Determination of their Source. By **Major W. D. Sutherland,** M.D., of His Majesty's Indian Medical Service. Small octavo, pp. 167. Illustrated. New York: William Wood & Co. (Cloth, $2.75.)

The medical and the legal professions both are appealed to by the contents of this brochure. It is not easy to determine whether a particular stain is due to blood or other sources, and it may be of the utmost importance to determine differentially this fact; a human life may poise in the balance until it is settled; a murderer may be brought to justice by the decision. No similar work in any language has appeared and it has remained for this author, a British military medical officer, to supply the deficiency. Major Sutherland has performed his self-imposed task with scientific fidelity to truth; he has given the modern tests for the detection of blood-stains; he has supplied invaluable information for the legal and medical professions. The precipitin test here receives the attention its merits deserve; while the alexine-fixation test, which is new, is discussed with definite knowledge of its true value. This is one of the most useful monographs that has issued from the press within recent years.

---

**A Textbook of Embryology.** By **John C. Heisler,** M.D., professor of Anatomy in the Medico-Chirurgical College of Philadelphia. Third edition. Octavo, 432 pages, with 212 illustrations, 32 in colors. Philadelphia and London: W. B. Saunders Company, 1907. (Cloth, $3.00; half morocco, $4.25 net prices.)

As in most new editions of medical works, this one has been thoroughly revised, some of the text being rewritten, and other portions changed, amplified, or eliminated according to the conditions governing the section under consideration. A revival of interest in embryology in recent years has lead to an increase in the literature of the subject bringing out a few new facts; but Heisler, who was a pioneer in this field of revival, has presented the topic in its most recent light, with all the known facts correlated in a fashion to make them available to the student of this too little explored field of research. The favorable reception of the first edition will, we doubt not, be extended to this later and more complete effort of this distinguished author.

---

**Hospital Training School Methods and the Head Nurse.** By **Charlotte A. Aitkens,** late Director of Sibley Memorial Hospital, Washington, D. C. 12mo, pp. 267. Philadelphia and London: W. B. Saunders Co. (Cloth, 1.50.)

Nurses everywhere and of all persuasions will profit by a study of this book. It instructs in the teaching of nurses; it teaches how to teach; it tells the nurse not only what to do and how it should be done, but also what not to do. It, also, gives valuable information for the head nurse in her capacity as such. If some head nurses we know would study this book and heed its counsels, they would become more useful in their responsible positions. Head nurses should have a clear conception of their relations to the institutions that employ them, to the officers, patients, physicians and all others who have any duties to perform in the hospitals. This book will help to give such a nurse a better status; it will help her to keep the place she has undertaken to fill; it is a pioneer in its chosen field and is a credit to its author.

---

**The Practice of Obstetrics.** By American Authors. Edited by **Charles Jewett**, M.D., Professor of Obstetrics in the Long Island College Hospital, Brooklyn, N. Y. Octavo, pp. 786, with 455 engravings in black and colors and 36 full-page colored plates. New York and Philadelphia: Lea Brothers & Co. (Cloth, $5.00; leather, $6.00; half morocco, $6.50 net prices.)

From the first this work took a high position in the medical world, particularly among obstetricians, and it has maintained its status with ever increasing favor. The editor had distinguished himself as a teacher of obstetrics long before he became an author, and he has surrounded himself in creating this work with a staff of well-known obstetricians as well as some other writers of distinction. This third edition has been extensively revised, much of it having been rewritten, until it now represents the most modern views on all obstetric questions. It is one of the best guides for the everyday practitioner, who must needs know his obstetrics well in order to meet the emergency of an active calling, one that makes more sudden demands on the resources and physical endurance of a physician than any other branch of medical practice. It is admirably illustrated and fills all the requirements of a modern obstetrical treatise.

---

**Clinical Lectures on Neurasthenia.** By **Thomas D. Savill**, M.D., physician to the West-End Hospital for Diseases of the Nervous System, London. Small 8 vo., pp. 216. New York: William Wood and Company, 1907. (Price, $1.50.)

These lectures have received the favor of the profession and may be regarded as the most scientific exposition of the phenomena to which we have attached a name that cloaks a multitude of ills which are greatly misunderstood. Functional disturbances of the nervous system are destined to furnish opportunity for empirical treatment, but gradually light is being turned upon much that has been mysterious, by such men as Savill, and we may hope, soon or late, to have a clearer solution of many dark or doubtful conditions.

This book affords pleasant reading, the author's syntax being clear and his rhetoric charming. We commend the plan of dating the prefaces to the several editions. If authors would always do this it would serve to maintain a sequence of valuable historical data.

---

**Nephritis.** A Manual of Bright's Disease and of Allied Disorders of the Kidneys, by Seelye W. Little, M.D. 12 mo., pp. 134. New York: The Grafton Press. 1907. (Price, $1.25.)

The importance of acquiring knowledge concerning diseases of the kidney was never so great as at the present moment. Our daily methods and habits of living and working would seem to provoke kidney break down at an earlier period of life than during the time when movements of men and things were slower. This excellent monograph, written in happy style, will aid in the solution of some of the difficult problems relating to nephritis. It tells of some valuable things to do as well as of many which should not be done. It gives valuable instruction relating to foods and dietaries, and every practising physician will find it agreeable reading. The author will one day find himself famous if he continues on the lines indicated in this book.

---

**A Pocket Textbook of Materia Medica, Therapeutics, Prescription Writing, Medical Latin and Medical Pharmacy.** By **William Schleif,** Ph.G., M.D., University of Pennsylvania, Philadelphia. Third edition, 12mo, 470 pages. Philadelphia and New York: 1907. Lea Brothers & Co. (Cloth, $2.50 net.).

This handsome volume, now in its third edition, is an excellent condensation of materia medica and therapeutics. It also takes under consideration a wide range of cognate subjects that are discussed with clearness. The text has been revised with care and made to conform to the new pharmacopeia, and nearly one hundred pages have been added. It is one of the better of the small works and covers the entire field of materia medica in a practical manner.

---

**Laboratory Manual of Invertebrate Zoology.** By **Gilman A. Drew,** Ph.D., Professor of Biology at the University of Maine. 12 mo, pp. 201. Philadelphia and London: W. B. Saunders Company. 1907. (Price, $1.25.)

The interesting study of the invertebrates will be facilitated by the use of this manual. It will stimulate to the acquirement of a more perfect knowledge of comparative anatomy, and should lead to an appreciation of the adaptations, wonderful in form and number, that fit the particular lives of different animals. In the study of biology it is an important book, and will prove a help to research in this essential topic of preliminary medical equipment.

